# AKR1D1 is a novel regulator of metabolic phenotype in human hepatocytes and is dysregulated in non-alcoholic fatty liver disease

**DOI:** 10.1016/j.metabol.2019.153947

**Published:** 2019-10

**Authors:** Nikolaos Nikolaou, Laura L. Gathercole, Lea Marchand, Sara Althari, Niall J. Dempster, Charlotte J. Green, Martijn van de Bunt, Catriona McNeil, Anastasia Arvaniti, Beverly A. Hughes, Bruno Sgromo, Richard S. Gillies, Hanns-Ulrich Marschall, Trevor M. Penning, John Ryan, Wiebke Arlt, Leanne Hodson, Jeremy W. Tomlinson

**Affiliations:** a Oxford Centre for Diabetes, Endocrinology and Metabolism, NIHR Oxford Biomedical Research Centre, University of Oxford, Churchill Hospital, Oxford OX3 7LE, UK; b Department of Biological and Medical Sciences, Oxford Brookes University, Oxford OX3 0BP, UK; c Translational Gastroenterology Unit, University of Oxford, Oxford, UK; d Institute of Metabolism and Systems Research, University of Birmingham, Edgbaston, Birmingham B15 2TT, UK; e Department of Upper GI Surgery, Churchill Hospital, Oxford University Hospitals NHS Foundation Trust, Oxford, UK; f Department of Molecular and Clinical Medicine, Institute of Medicine, University of Gothenburg, 413 45 Gothenburg, Sweden; g Department of Systems Pharmacology & Translational Therapeutics, University of Pennsylvania Perelman School of Medicine, 1315 BRB II/III 421 Curie Blvd, Philadelphia, PA 19104-6160, United States of America

**Keywords:** NAFLD, 5β-Reductase, Diabetes, Bile acids, Triglyceride, FXR

## Abstract

**Objective:**

Non-alcoholic fatty liver disease (NAFLD) is the hepatic manifestation of metabolic syndrome. Steroid hormones and bile acids are potent regulators of hepatic carbohydrate and lipid metabolism. Steroid 5β-reductase (AKR1D1) is highly expressed in human liver where it inactivates steroid hormones and catalyzes a fundamental step in bile acid synthesis.

**Methods:**

Human liver biopsies were obtained from 34 obese patients and AKR1D1 mRNA expression levels were measured using qPCR. Genetic manipulation of AKR1D1 was performed in human HepG2 and Huh7 liver cell lines. Metabolic assessments were made using transcriptome analysis, western blotting, mass spectrometry, clinical biochemistry, and enzyme immunoassays.

**Results:**

In human liver biopsies, *AKR1D1* expression decreased with advancing steatosis, fibrosis and inflammation. Expression was decreased in patients with type 2 diabetes. In human liver cell lines, *AKR1D1* knockdown decreased primary bile acid biosynthesis and steroid hormone clearance. RNA-sequencing identified disruption of key metabolic pathways, including insulin action and fatty acid metabolism. *AKR1D1* knockdown increased hepatocyte triglyceride accumulation, insulin sensitivity, and glycogen synthesis, through increased *de novo* lipogenesis and decreased β-oxidation, fueling hepatocyte inflammation. Pharmacological manipulation of bile acid receptor activation prevented the induction of lipogenic and carbohydrate genes, suggesting that the observed metabolic phenotype is driven through bile acid rather than steroid hormone availability.

**Conclusions:**

Genetic manipulation of AKR1D1 regulates the metabolic phenotype of human hepatoma cell lines, driving steatosis and inflammation. Taken together, the observation that *AKR1D1* mRNA is down-regulated with advancing NAFLD suggests that it may have a crucial role in the pathogenesis and progression of the disease.

## Highlights

• AKR1D1 expression is downregulated in patients with non-alcoholic fatty liver disease
• AKR1D1 regulates bile acid availability in human hepatocytes
• AKR1D1 silencing drives insulin sensitivity, lipid accumulation and fuels hepatocyte inflammation
• Alterations in FXR and LXR activation underpin some, but not all, of the metabolic effects of AKR1D1 silencing

## 1 Introduction

The global burden of metabolic disease, including obesity, type 2 diabetes mellitus (T2DM) and its hepatic manifestation, non-alcoholic fatty liver disease (NAFLD), continues to escalate. NAFLD is common, with an unselected population prevalence of 30%, rising to over 80% in obese T2DM patients. It is a spectrum of disease ranging from simple steatosis through to inflammation (non-alcoholic steatohepatitis, NASH) and eventual scarring, fibrosis and cirrhosis with a significant risk of development of hepatocellular carcinoma (HCC) alongside increased cardiovascular mortality [1]. NAFLD is rapidly becoming the leading indication for liver transplantation [2]. Despite the magnitude of the clinical problem, defining the precise molecular pathways that drive lipid accumulation and have potential as therapeutic targets have proved complex.

Steroid hormones, including glucocorticoids and androgens, are potent regulators of metabolic phenotype and have been implicated in driving hepatic lipid accumulation. Glucocorticoid excess, androgen deficiency in men, and androgen excess in women are all associated with NAFLD [[3], [4], [5]]. Steroid hormone availability is regulated at pre-receptor level within the liver by a series of enzymes, and both genetic and pharmacological manipulation of these enzymes has a profound impact upon metabolic phenotype, including the development of hepatic steatosis [[6], [7], [8]]. Steroid 5β-reductase (AKR1D1) has an important pre-receptor role in the regulation of steroid hormone action, however, its role in regulating metabolic phenotype has not been explored to date [9]. AKR1D1 is highly expressed in human liver where it generates all 5β-reduced dihydrosteroid metabolites for C19-, C21- and C27-steroids, *i.e.* androgens, glucocorticoids, and bile acids [10]. In addition to its role in steroid hormone clearance, AKR1D1 has a crucial function in bile acid synthesis. Bile acids are now established as potent signalling molecules that regulate metabolic phenotype acting through previously orphan nuclear receptors, including the farnesoid-X-receptor (FXR). In addition, oxysterols, including bile acid intermediary substrates, are able to also activate the liver-X receptors α and β (LXRα, LXRβ) [11]. The activation of both FXR and LXRs have been implicated in the pathogenesis of NAFLD. FXR deletion results in hepatic steatosis, insulin resistance and hyperglycaemia, with evidence suggesting decreased expression in NAFLD [12,13], and FXR agonism has already shown early promise as a therapeutic strategy in NAFLD [14]. In contrast, LXRα expression is increased in NAFLD [11] and LXRα activation drives lipid accumulation whilst suppressing the local inflammatory response [15].

AKR1D1 sits at the interface of two fundamentally important pathways, steroid hormone metabolism and bile acid synthesis, both being potent regulators of hepatic metabolic phenotype. The putative role of AKR1D1 in this regard is almost entirely unexplored; a single study found an association between increased systemic 5β-reductase activity and hepatic steatosis [16], another demonstrated decreased hepatic 5β-reductase expression in diabetic patients [17], whilst a third study reported elevated levels of 7-alpha-hydroxy-4-cholesten-3-one, the bile acid precursor substrate for AKR1D1, in patients with NAFLD [18].

We have therefore hypothesised that decreases in AKR1D1 activity and expression will have a detrimental impact upon hepatic metabolic phenotype, either through decreased bile acid availability or impaired steroid hormone clearance. We have tested this hypothesis through genetic manipulation of *AKR1D1* gene expression as well as investigating expression in human liver biopsies with established NAFLD.

## 2 Material and methods

### 2.1 Human studies

Obese male (*n* = 7) and female (*n* = 27) individuals undergoing primary bariatric surgery (laparoscopic sleeve gastrectomy or Roux-en-Y gastric bypass) were eligible to be recruited into our dedicated bariatric surgery biobank study (Ref: 16/YH/0247) subject to their written informed consent. Detailed clinical data (including weight, height, medical history, medications, family history and alcohol intake) was recorded for all participants prior to surgery. Intraoperatively, a wedge biopsy was taken from the anatomical left lobe of the liver after pneumoperitoneum was established. Half of this tissue was stored in formalin prior to histological analysis by a consultant liver histopathologist according to Kleiner's NAFLD Activity Score (NAS) and fibrosis staging classification [19]; the remainder was washed in 0.9% NaCl then stored in RNAlater stabilization solution at −80 °C until RNA extraction.

### 2.2 Cell culture

HepG2 cells were a gift from Dr. Karl Morten (University of Oxford), HEK293 cells were a gift from Prof. Anna Gloyn (University of Oxford), Hep3b cells were a gift from Prof Jane McKeating (University of Birmingham) and Huh7 cells were a gift from Dr. Camilla Pramfalk (Karolinska Institutet). All cells were cultured in Dulbecco's Minimum Essential Medium (DMEM) (Zen Bio Inc., Durham, NC, USA), containing 4.5 g/L glucose, and supplemented with 10% fetal bovine serum, 1% penicillin/streptomycin and 1% non-essential amino acids.

The C3A human hepatocyte cell line was purchased from LGC Standards (ATCC - CRL-10741, Middlesex, UK), and cultured in Eagle's Minimum Essential Medium (EMEM) (Sigma-Aldrich, Dorset, UK) containing 4.5 g/L glucose, and supplemented with 10% fetal bovine serum, 1% penicillin/streptomycin and 1% non-essential amino acids.

### 2.3 Primary human hepatocytes

Liver was obtained from patients undergoing surgery who had consented (NRES Committee South Central; Berkshire B 11/SC/0443) to the use of excess tissue (resection surplus) for research. Hepatocytes were dissociated from whole liver tissue *via* a two-stage collagenase digestion as previously described [20]. Cells were diluted to 1 million cells per mL in William's E buffer containing hepatocyte supplements before plating on Type I collagen coated plates. Cells were collected 24 h post seeding for RNA isolation.

### 2.4 Transfection studies

For over-expression transfection studies, 1.6 × 10^5^ cells/well were plated in 12-well Cell Bind plates (CORNING, Flintshire, UK) for 24 h prior to transfection with the pCMV6-XL4 + AKR1D1 construct (Origene Technologies, Rockville, USA). 1 μg DNA construct and 2 μL X-tremeGENE DNA transfection Reagent (Roche, Hertfordshire, UK) were diluted in 100 μL OPTIMEM serum-free media (Invitrogen, Paisley, UK). The mixture was vortexed and incubated at room temperature for 20 min and, subsequently, 100 μL was added to each well and cells incubated at 37 °C for 48 h prior to treatment.

For knockdown transfection studies, 0.8 × 10^5^ cells/well were plated in 24-well Cell Bind plates (CORNING, Flintshire, UK) for 24 h prior to transfection. To prepare siRNA/lipid solutions for each well of a 24-well plate, 20 nmol of AKR1D1 siRNA (Invitrogen, Paisley, UK) was diluted in 25 μL of OPTIMEM serum free media (Invitrogen, Paisley, UK). In a separate tube, 2.5 μL of Lipofectamine RNAiMAX (Invitrogen, Paisley, UK) was diluted in 25 μL of OPTIMEM. The contents of the two tubes were combined by gentle pipetting and incubated at room temperature for 5 min. The resulting 50 μL of transfection solution was added drop-wise. Finally, cells were then incubated at 37 °C for 48 h.

### 2.5 Cell treatments

Cortisol, cortisone, cholic acid (CA), chenodeoxycholic acid (CDCA), GW4064, 22(*S*)-Hydroxycholesterol and GSK2033 were purchased from Sigma-Aldrich (Dorset, UK). All cell treatments, including bile acid measurements, were performed in serum-free and phenol-red free media, containing 4.5 g/L glucose (Zen Bio Inc., Durham, NC, USA).

### 2.6 Steroid hormone measurements

Cortisone was extracted from cell media after addition of the internal standard, cortisol-d4. Briefly, transfected cells were incubated with 200 nM of cortisone for 8 h. 1 mL of media was collected, extracted by SPE and the samples were derivatized overnight to form methyloxime-trimethylsilylethers (MO-TMS). The final derivative was dissolved in 55 μL cyclohexane, which was transferred to an auto-sampler vial for gas chromatography–mass spectrometry analysis using an Agilent 5973 instrument (www.agilent.com) in a selected ion monitoring mode.

### 2.7 Lipid extraction and gas chromatography–mass spectrometry

Total intracellular lipid was extracted and intracellular *de novo* lipogenesis (DNL) measured by the uptake of deuterium from ^2^H_2_O (Finnigan GasBench-II, ThermoFisher Scientific, UK) into intracellular triglyceride and PL [^2^H]-palmitate. [^2^H]-palmitate enrichments were determined by gas chromatography–mass spectrometry using a 5890 GC coupled to a 5973 N MSD (Agilent Technologies; CA, USA). Ions with mass-to-charge ratios (*m*/*z*) of 270 (M + 0) and 271 (M + 1) were determined by selected ion monitoring [21].

### 2.8 RNA extraction and reverse transcription

Total RNA was extracted from cells using the Tri-Reagent system (Sigma-Aldrich, Dorset, UK). Concentration was determined spectrophotometrically at OD260 on a Nanodrop spectrophotometer (Thermo Scientific, Hemel Hempstead, UK). Reverse transcription was performed in a 20 μL volume; 1 μg of total RNA was incubated with 10× RT Buffer, 100 mM dNTP Mix, 10× RT Random Primers, 50 U/μL MultiScribe Reverse Transcriptase and 20 U/μL RNase Inhibitor (Thermo Scientific, Hemel Hempstead, UK). The reaction was carried out at 25 °C for 10 min, 37 °C for 120 min and then terminated by heating to 85 °C for 5 min.

### 2.9 Quantitative PCR (qPCR)

All quantitative PCR experiments were conducted using an ABI 7900HT sequence detection system (Perkin-Elmer Applied Biosystems, Warrington, UK). Reactions were performed in 6 μL volumes on 384-well plates in reaction buffer containing 3 μL of 2 x Kapa Probe Fast qPCR Master Mix (Sigma-Aldrich, Dorset, UK). Life Technologies supplied all primers as predesigned TaqMan Gene Expression Assays labelled with FAM. The reaction conditions were as follows: 95 °C for 3 min, then 40 cycles of 95 °C for 3 s and 60 °C for 20s. The Ct (dCt) of each sample using the following calculation (where E is reaction efficiency – determined from a standard curve): ΔCt = E^[min Ct-sample Ct]^ using the 1/40 dilution from a standard curve generated from a pool of all cDNAs as the calibrator for all samples. The relative expression ratio was calculated using the following: Ratio = ΔCt_[target]_/ΔCt_[ref]_ and expression values were normalised to 18SrRNA, unless stated otherwise [22].

### 2.10 RNA sequencing

Total RNA was extracted using the Tri-Reagent system (Sigma-Aldrich, Dorset, UK) and enriched for polyA-tailed mRNA using oligo (dT) beads. The Illumina TruSeq Stranded mRNA HT Sample Prep Kit was used to prepare cDNA libraries for sequencing. In-house 8 bp indexes [23] were used to multiplex samples (10-plex), which were then sequenced over 1 lane of an Illumina HiSeq4000 machine using HiSeq 3000/4000 PE Cluster Kit and SBS Kit. Paired-end sequencing (75 bp) was performed at a depth of ~25 million read pairs per sample. Reads were mapped with STAR 2.5.1b [24] on default settings with GENCODE version 19 [25] as transcriptome and GRCh37 as genome reference. Gene level reads counts for all protein-coding and long intergenic non-coding RNA (lincRNA) transcripts present in GENCODE version 19 were quantified in a strand-specific manner with featureCounts [26] from the Subread package v1.5.0-p2. For plotting purposes, we also normalised the gene counts to transcripts per million (TPM). Differential expression analysis was performed using limma [27] in R 3.2.2 on voom-normalised gene counts for all autosomal protein-coding and lincRNA genes that were expressed at >1 count per million (CPM) in all knockdown and/or all control samples. A paired model was fitted to the data, and significance was determined by empirical Bayes moderated t-statistics implemented in limma. Differentially regulated genes were defined by a false discovery rate (Benjamini-Hochberg method) adjusted *p*-value <1%.

### 2.11 Protein extraction and Immunoblotting

Total protein was extracted from cells using RIPA buffer (Sigma-Aldrich, Dorset, UK), protease inhibitor cocktail (1/100) and phosphatase inhibitor cocktail (1/100) (ThermoFisher Scientific, Loughborough, UK). Protein concentrations were measured using a commercially available assay according to the manufacturer's protocol (Bio-Rad, Hemel Hempstead, UK). Primary β-tubulin (#15115, monoclonal), total AKT (#4685, monoclonal), pAKT (#4060, monoclonal), AKT1 (#75692, monoclonal), mTOR (#2983, monoclonal), pGSK3β (#5558, monoclonal), GSK3β (#12456, monoclonal), ACC (#3662, polyclonal), pACC (#11818, monoclonal), IκΒα (#4812, monoclonal) from Cell Signalling (Danvers, USA), AKR1D1 (dilution 1/250) (HPA057002, polyclonal, Atlas Antibodies AB, Bromma, Sweden), and secondary antibodies (P044801–2, P044701–2, polyclonal) from Dako (Agilent Technologies, Santa Clara, USA) were used at a dilution of 1/1000 and 1/2000 respectively, unless stated otherwise. To ascertain equal gel loading, proteins were normalised to β-tubulin. Bands were visualised with ECL and ChemiDocXS imager (Biorad, Hemel Hempstead, UK). Western blots were quantified by densitometry analysis using Image J (NIH, Bethesda, MD, http://rsb.info.nih.gov/ij).

### 2.12 Bile acid measurements

Total bile acid content was measured using a colorimetric assay according to the manufacturer's protocol (Cell Biolabs Inc., San Diego, USA). Total CA and CDCA levels were measured using a competitive ELISA according to the manufacturer's protocol (Cell Biolabs Inc., San Diego, USA).

### 2.13 Biochemical measurements

Glucose, triglyceride and 3-hydroxybutyrate were analyzed using Instrumentation Laboratory kits on an ILab 650 Clinical Chemistry analyzer as described previously [28]. Complete medium-containing FBS or lysis buffer were used as negative background controls. Intracellular glycogen content was measured using a fluorimetric assay according to the manufacturer's protocol (BioVision Inc., California, USA).

### 2.14 Statistics

Data are presented as mean ± standard error. Normal distribution was confirmed using Shapiro-Wilk test. Statistical analysis on qPCR data from human subjects was performed using two-tailed, unpaired *t*-tests and Fisher's exact *t*-test. For cell culture experiments, two-tailed, paired *t*-tests were used to compare single treatments to control. For comparisons between control and different treatments, statistical analysis was performed using one-way analysis of variance (ANOVA) with Dunnett corrections. To compare mean differences between groups that had been split on multiple treatments, doses or times, two-way analysis of variance (ANOVA) with Sidak corrections was used. Statistical analysis on qPCR data was performed on mean relative expression ratio values. Data analysis was performed using Graphpad Prism software (Graphpad Software Inc., La Jolla, USA) and considered statistically significant at *p* < 0.05.

## 3 Results

### 3.1 AKR1D1 mRNA expression decreases across the spectrum of NAFLD in humans

**Fig. 1:**
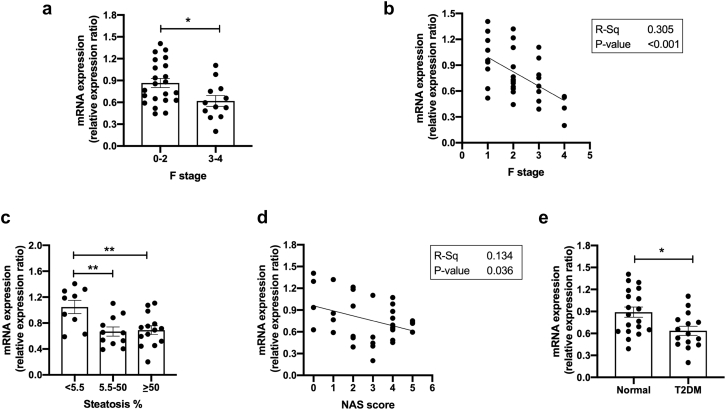
AKR1D1 expression in obese patients. In male (*n* = 7) and female (*n* = 27) obese patients, *AKR1D1* mRNA expression decreased with more advanced fibrosis (a-b), steatosis percentage (c) and higher NAFLD Activity Score (NAS) (d). In addition, mRNA expression of AKR1D1 was reduced in patients with T2DM (e). qPCR data were normalised to 18SrRNA, *TBP* and *ACTB*. Data are presented as mean ± se, performed in triplicate, *p < 0.05, **p < 0.01.

AKR1D1 mRNA levels were examined in liver biopsies from obese male and female patients (*n* = 34). A summary of patient characteristics is provided in Table 1. *AKR1D1* expression was significantly lower in advanced fibrosis (F3–4), when compared to mild to moderate fibrosis (F0–2) (Fig. 1a and b). When biopsies were categorized on the basis of NAFLD activity score (NAS), reflecting the presence of non-alcoholic steatohepatitis (NASH), as well as on the basis of steatosis percentage, AKR1D1 mRNA levels were lower in those with higher NAS and > 5.5% steatosis (the diagnostic threshold for hepatic steatosis [29,30]) (Fig. 1c–d). In addition, when patients were categorized based on the presence of T2DM, AKR1D1 mRNA levels were significantly lower in patients with T2DM (Fig. 1e). Endorsing our findings, additional data from publicly available databases were extracted, demonstrating a consistent decrease in *AKR1D1* expression in patients with advanced NAFLD, compared to mild NAFLD (Supplementary Fig. 1) (GEO accession GSE49541; [31,32]).

**Table 1:** Clinical and biochemical characteristics of obese patients with mild to moderate fibrosis (F0–2) compared to patients with advanced fibrosis (F3–4) on liver biopsy.

Characteristics	F score		p-Value
	0–2	3–4	
Gender (m/f)	f = 21, m = 1	f = 6, m = 6	
Age (years)	45 ± 3	49 ± 3	0.32
BMI (kg/m^2^)	48.8 ± 1.7	48.0 ± 2.3	0.788
T2DM (%)	18 (4/22)	92 (11/12)***	<0.001
AST (IU/L)	22 ± 1	32 ± 5*	0.016
ALT (IU/L)	29 ± 2	37 ± 7	0.167
Platelets (x10^9^/L)	269 ± 11	239 ± 23	0.198
AST/ALT ratio	0.85 ± 0.05	0.92 ± 0.11	0.569
FIB-4 score	0.76 ± 0.08	1.55 ± 0.36**	0.007
Ferritin (ug/L)	102 ± 13	120 ± 20	0.448
HbA1c (%)	5.5 ± 0.1	6.4 ± 0.4**	0.004
Total Chol (mmol/L)	4.6 ± 0.3	4.0 ± 0.3	0.16
HDL (mmol/L)	1.0 ± 0.05	0.8 ± 0.03**	0.008
LDL (mmol/L)	2.8 ± 0.2	2.3 ± 0.3	0.178
Chol/HDL ratio	4.6 ± 0.2	4.8 ± 0.3	0.597

To define whether the observed differences in *AKR1D1* expression are sex-specific, mRNA analysis was repeated in female subjects only. Consistent with the previous data, in obese female patients, *AKR1D1* expression was significantly lower in advanced fibrosis (F0–1: 0.85 ± 0.06 *vs.* F3–4: 0.59 ± 0.09, *p* = 0.05) and in patients with T2DM (Normal: 0.90 ± 0.07 *vs.* T2DM: 0.60 ± 0.06, *p* < 0.01).

### 3.2 *AKR1D1* silencing decreases steroid hormone clearance and bile acid synthesis in human hepatoma cells

In order to investigate the role of AKR1D1 within the human liver *in vitro*, the expression pattern of *AKR1D1* expression was explored in four different human hepatoma cell lines, using qPCR (Supplementary Fig. 2a). Expression levels in HepG2 and Huh7 were similar to those seen in primary human hepatocytes and these were therefore selected as suitable models for genetic manipulation studies.

**Fig. 2:**
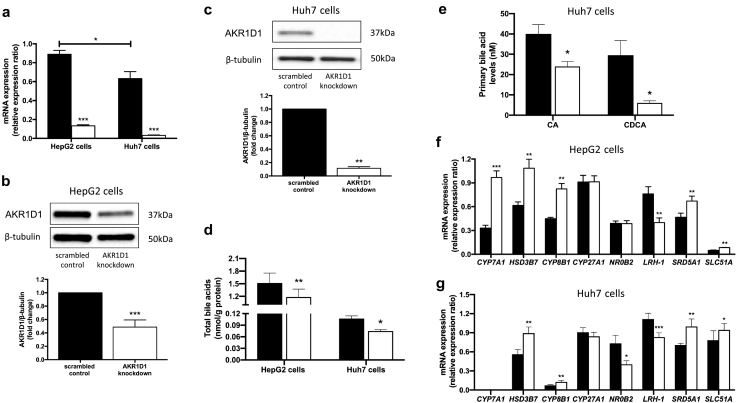
Genetic manipulation of AKR1D1 in human hepatoma cell lines and bile acid signalling and synthesis in human hepatoma cell lines, following *AKR1D1* knockdown. *AKR1D1* knockdown (white bars), decreased mRNA and protein expression in both HepG2 and Huh7 cells, as measured by qPCR and western blotting (a-c). In both HepG2 and Huh7 cells, *AKR1D1* knockdown (white bars) decreased total bile acid concentrations (d). In addition, *AKR1D1* knockdown decreased cholic acid (CA) and chenodeoxycholic acid (CDCA) concentrations in Huh7 cells (e). Consistent with these, *AKR1D1* knockdown increased the mRNA expression of genes involved in classic bile acid synthesis pathway (*CYP7A1*, *HSD3B7*, *CYP8B1*), bile acid transport (*SLC51A*), and FXR activation (*NR0B2*, *LRH*-*1*) (f-g). Representative Western blot images are shown, however formal quantification was performed in n = 7–9 replicates. qPCR data were normalised to 18SrRNA. Data are presented as mean ± se of *n* = 5–8 experiments, performed in triplicate, *p < 0.05, **p < 0.01, ***p < 0.001, compared to scrambled controls (black bars).

siRNA techniques were utilised in both cell lines to decrease AKR1D1 mRNA levels and effective knockdown was confirmed by qPCR and western blotting (Fig. 2a–c). To confirm the functional impact of *AKR1D1* knockdown on glucocorticoid clearance, HepG2 cells were treated with cortisone (200 nM, 8 h) and cell culture media cortisone and 5β-tetrahydrocortisone (5β-reduced metabolite of cortisone, 5β-THE) levels were measured using GC–MS, and compared against scrambled controls. Consistent with the mRNA and protein data, *AKR1D1* knockdown decreased cortisone clearance and 5β-THE generation (Supplementary Fig. 2b and c).

Following over-expression of *AKR1D1* in HepG2 and HEK293 cells, cortisone clearance and 5β-THE generation were increased, providing additional evidence for the potent ability of AKR1D1 to regulate steroid hormone availability within human hepatoma cells (Supplementary Figs. 2d–g and 3a–d).

As AKR1D1 plays a crucial role in bile acid synthesis, we examined cell culture media bile acid levels following *AKR1D1* knockdown in both HepG2 and Huh7 cells, revealing decreased total bile acid secretion in the cell culture media (Fig. 2d). Specifically, cell media CA and CDCA levels were reduced (Fig. 2e). In both cell lines, compensatory increases in the mRNA expression of genes involved in primary bile acid synthesis and transport, including *CYP7A1*, *HSD3B7*, *CYP8B1* and *SLC51A*, were observed, in addition to decreases in the expression of the FXR regulated genes *NR0B2* (*SHP*) and *LRH*-*1*, indicating decreased FXR activation (Fig. 2f and g). Interestingly, a significant increase in the mRNA expression of 5α-reductase type 1 (*SRD5A1*) was also observed (Fig. 2f and g), suggesting a compensatory mechanism for either steroid clearance or bile acid production.

### 3.3 *AKR1D1* silencing defines discrete dysregulated metabolic pathways

**Fig. 3:**
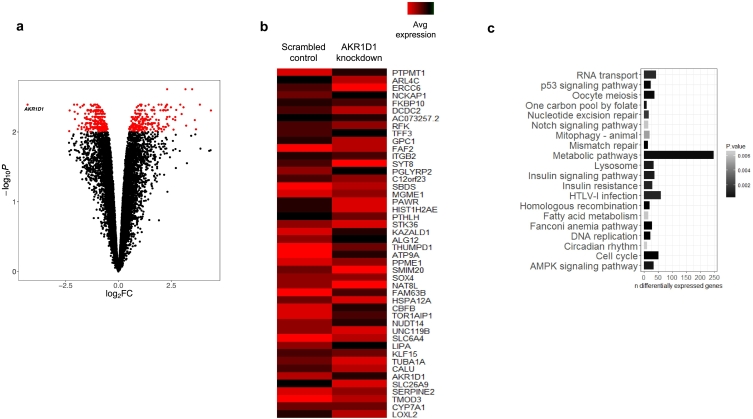
RNA sequencing analysis in HepG2 cells, following AKR1D1 knockdown. Transcriptomic profiling of AKR1D1 knockdown in HepG2 cells. Volcano plot illustrating fold-change in expression (log_2_FC) against statistical significance (–log_10_ adjusted *P*-values) for all genes. Red dots represent differentially expressed genes as a function of *AKR1D1* knockdown (FDR corrected P-values <0.01) (a). Heat map of the expression changes (normalised counts per million) of the top 10% differentially expressed genes (FDR 1%) in *AKR1D1* knockdown HepG2 cells (red and black bars represent up- and down-regulation, respectively) (b). Top KEGG pathways illustrated as a function of the number of differentially expressed genes contributing to each annotated pathway and coloured with a gradient defined by the statistical significance of pathway enrichment (c).

To gain a broader insight into the role of AKR1D1 in human hepatoma cells, transcriptome analysis using RNA-sequencing was undertaken in HepG2 cells with AKR1D1 knockdown. A total of 465 transcripts were differentially expressed as a function of *AKR1D1* knockdown (*p* = 4.06 × 10^−3^; logFC = −4.29) compared to scrambled control-transfected HepG2 cells. The top 10% of differentially regulated genes are presented in the heatmap in Fig. 3b. To facilitate wider exploration of molecular processes affected by *AKR1D1* knockdown, a differential expression gene set obtained with a more relaxed significance threshold (FDR 5%) was used to search for enrichment of biological pathways annotated in the Kyoto Encyclopaedia of Genes and Genomes (KEGG). This analysis identified discrete dysregulated metabolic pathways impacting upon a variety of cellular phenotypes, notably, including key pathways impacting insulin action and fatty acid storage and utilization (Fig. 3c).

### 3.4 *AKR1D1* knockdown enhances insulin sensitivity in human hepatoma cells

**Fig. 4:**
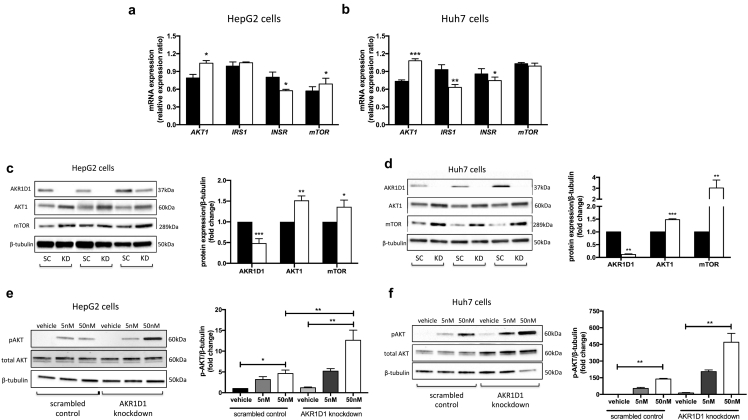
Insulin signalling in human hepatoma cell lines, following AKR1D1 knockdown. *AKR1D1* knockdown (white bars) altered the mRNA expression of insulin signalling genes, including AKT1, insulin receptor and mTOR, in both HepG2 (a) and Huh7 (b) cells, as measured by qPCR. *AKR1D1* knockdown (white bars) increased the protein expression of both AKT1 and mTOR in both cell lines (c and d). Consistent with these data, AKR1D1 knockdown, enhanced insulin sensitivity with increased insulin-stimulated phosphorylation of AKT and mTOR (15 min treatment), in both HepG2 (e) and Huh7 (f) cells. qPCR data were normalised to 18SrRNA and protein data were normalised to β-tubulin. Representative Western blot images are shown from 3 biological replicates, however formal quantification was performed in n = 7–9 replicates. Gene expression data are presented as mean ± se of n = 7 experiments, performed in triplicate, *p < 0.05, **p < 0.01, ***p < 0.001, compared to scrambled controls (black bars).

Informed by our RNA sequencing data, we undertook a series of experiments to define the precise impact of *AKR1D1* silencing on hepatic insulin sensitivity *in vitro*. In both HepG2 and Huh7 cells, *AKR1D1* knockdown increased the mRNA expression of genes involved in insulin signalling, including *AKT1* and *mTOR*, whilst it decreased insulin receptor expression (*INSR*) (Fig. 4a and b). Consistent with these findings, *AKR1D1* knockdown increased the protein expression of AKT1 and mTOR in both human hepatoma cell lines, indicative of alterations in the activation of the insulin signalling cascade (Fig. 4c and d). Endorsing the changes in protein expression, insulin-stimulated phosphorylation of PKB/akt at serine 473 was increased in both HepG2 and Huh7 cells following *AKR1D1* knockdown, compared to scrambled controls, in a dose-dependent manner (5 nM and 50 nM, 15 min) (Fig. 4e and f).

### 3.5 *AKR1D1* knockdown drives hepatocyte triglyceride accumulation through enhanced lipogenesis and decreased oxidation

**Fig. 5:**
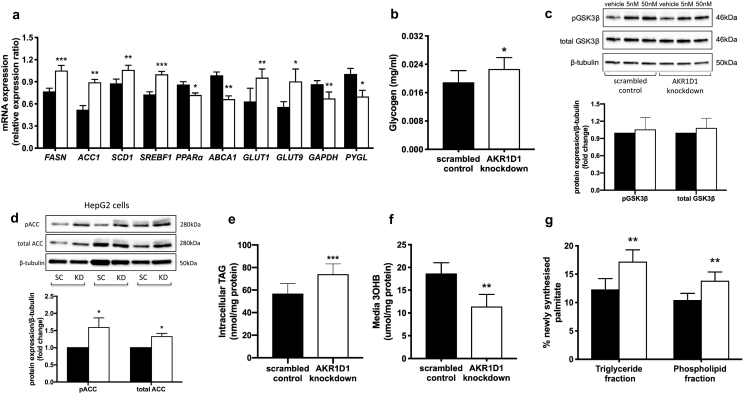
Lipid and carbohydrate metabolism in human hepatoma cell lines, following AKR1D1 knockdown. *AKR1D1* knockdown (white bars) increased *GLUT1* and *GLUT9* and decreased *GAPDH* and glycogen phosphorylase (*PYGL*) mRNA expression in HepG2 cells, as measured by qPCR (a). Furthermore, intracellular glycogen storage increased (b), whilst, no differences were observed in total or phosphorylated GSK3β levels, following 15 min insulin stimulation (5 nM, 50 nM) (c). In addition, *AKR1D1* knockdown enhanced lipogenic gene expression (*FAS*, *ACC1*, *SCD1*, *SREBF1*) (a), reduced the expression of *ABCA1* and *PPARα* (a), and increased total and phosphorylated ACC protein levels (d). Consistent with the lipogenic and PPARα gene expression, *AKR1D1* knockdown increased TAG accumulation in HepG2 cells (e) and reduced fatty acid oxidation, as measured by cell media 3-hydroxybutyrate [3OHB] concentrations (f). Moreover, *AKR1D1* knockdown increased *de novo* palmitate synthesis in both TAG and phospholipid fraction, as measured by incorporation of ^2^H from ^2^H_2_O into TAG and phospholipid palmitate, respectively (g). Representative Western blot images are shown from 3 biological replicates, however formal quantification was performed in n = 7–9 replicates. qPCR data were normalised to 18SrRNA and protein data were normalised to β-tubulin. Data are presented as mean ± se of n = 7–8 experiments, performed in triplicate, *p < 0.05, **p < 0.01, ***p < 0.001, compared to scrambled controls (black bars).

Metabolic assessments were made in HepG2 cells following *AKR1D1* knockdown. mRNA expression of the glucose transporters GLUT1 and GLUT9 increased, whilst glyceraldehyde-3-phosphate dehydrogenase (*GAPDH*) and glycogen phosphorylase expression (*PYGL*) decreased (Fig. 5a). Consistent with these findings, cell media glucose concentrations were significantly lower (scrambled control: 15.34 ± 1.51 *vs.* AKR1D1 knockdown: 12.11 ± 0.90 nmol/mg, *p* < 0.05) and intracellular glycogen storage increased (Fig. 5b). However, no differences in the protein levels of either total or phosphorylated GSK3β were observed, suggesting that the differences in glycogen accumulation are likely to be due to decreased glycogenolysis rather than increased glycogen synthesis (Fig. 5c). In addition, we observed significant increases in lipogenic gene expression [fatty acid synthase (*FASN*), acetyl co-A carboxylase (*ACC1*), stearoyl-CoA desaturase 1 (*SCD1*) and sterol regulatory element-binding protein 1c (*SREBF1*)] and reductions in the expression of enzymes regulating lipid export [ATP-binding cassette subfamily A1 (*ABCA1*)] and oxidation [peroxisome proliferator-activated receptor α (PPARα)] (Fig. 5a). The pattern of changes in gene expression mirrored that observed in the RNA-sequencing analysis (Supplementary Fig. 4).

Consistent with the mRNA expression data, there were increased phosphorylated and total protein levels of ACC in *AKR1D1* knockdown HepG2 cells (Fig. 5d). Total cellular triglyceride (TAG) accumulation also increased (scrambled control: 56.9 ± 8.8 *vs. AKR1D1* knockdown: 74.1 ± 9.0 nmol/mg, *p* < 0.001), whilst fatty acid oxidation decreased, as measured by reduced 3-hydroxybutyrate concentrations in the cell culture media (Fig. 5e and f). TAG accumulation in hepatocytes can occur *via* a number of different pathways. *De novo* lipogenesis (DNL) is the synthesis of fatty acids, namely palmitate (16:0), from non-lipid precursors and this was measured by incorporation of deuterated water into cellular TAG and phospholipids. *AKR1D1* knockdown increased DNL both in total cellular TAG and in the phospholipid fraction (Fig. 5g). Complementary metabolic assessments were performed in Huh7 cells; the results revealed similar changes with increased lipogenic gene expression, increased ACC protein levels and increased TAG accumulation, following *AKR1D1* knockdown (Supplementary Fig. 5).

Parallel metabolic assessments were made in HepG2 cells over-expressing *AKR1D1*. However, no differences in the expression of genes involved in carbohydrate and lipid metabolism or intracellular TAG accumulation were observed (Supplementary Fig. 6).

### 3.6 Metabolic effects of *AKR1D1* knockdown are dependent upon reduced FXR and increased LXR activation

**Fig. 6:**
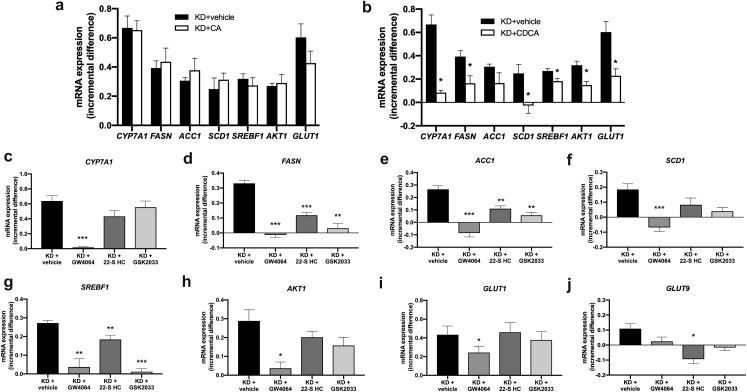
Incremental gene expression following *AKR1D1* knockdown followed by subsequent treatment with CA, CDCA, FXR agonst or LXR antagonist treatment to attempt to rescue the cellular phenotype. CA replacement (50 μM, 24 h) in the cell culture media failed to prevent upregulation of carbohydrate, lipid or insuling signalling gene expression in *AKR1D1* knocked down HepG2 cells (a). In contrast, CDCA replacement (50 μM, 24 h) significantly impaired upregulation of the expression of *CYP7A1*, *FASN*, *SCD1*, *SREBF1*, *AKT1* and *GLUT1*, in *AKR1D1* knocked down HepG2 cells (b). Pharmacological manipulation of the bile acid receptor FXR, using the FXR agonist GW4064 (5 μM, 24 h), also normalised the expression of *CYP7A1*, *FASN*, *ACC1*, *SREBF1*, *SCD1*, *AKT1*, *GLUT1* and *GLUT9* to levels seen in scrambled controls (figure c–h). In addition, treatment with the LXR antagonist 22-S Hydroxycholesterol (22-S HC - 10 μM, 24 h) and the LXRβ antagonist GKS2033 (100 nM, 24 h) partially restored the expression levels of *FASN*, *ACC1* and *SREBF1*, but had no impact on the induction of expression of the other genes caused by *AKR1D1* knockdown (c–h). qPCR data were normalised to 18SrRNA. Data are presented as mean ± se of incremental gene expression of *n* = 3–6 experiments, performed in triplicate, *p < 0.05, **p < 0.01, ***p < 0.001 and compared to vehicle treated controls. (KD = AKR1D1 knockdown).

*AKR1D1* knockdown significantly increased the expression of *CYP7A1*, *FASN*, *ACC1*, *SREBF1*, *SCD1*, *AKT1*, *GLUT1* and *GLUT9* in human hepatoma cells. Cortisol treatment (500 nM, 24 h) of HepG2 cells failed to regulate the mRNA expression of these genes, suggesting that the phenotype we observed in our *AKR1D1* knockdown experiments was unlikely to be due to changes in glucocorticoid availability (Supplementary Fig. 7). Additional treatments with CA (50 μM, 24 h) and CDCA (50 μM, 24 h) were performed. Whilst CA had no effect on gene expression, CDCA treatment significantly prevented the induction of *CYP7A1*, *FASN*, *SCD1*, *SREBF1*, *AKT1* and *GLUT1* mRNA expression caused by *AKR1D1* knockdown (Fig. 6a and b).

We therefore tested the hypothesis that the observed phenotype could arise from decreased FXR activation (due to decreased bile acid synthesis and availability) or increased LXR activation secondary to increased accumulation of oxysterols and bile acid precursors. We proposed that FXR agonism and/or LXR antagonism would have the potential to rescue the phenotype in our cells. The FXR agonist GW4064 (5 μM, 24 h) and the LXRα antagonist 22(*S*)-Hydroxycholesterol (22-S HC) (10 μM, 24 h) decreased *CYP7A1*, *GLUT1*, *FASN* and *SCD1* expression, whilst the LXRα antagonist selectively decreased *SREBF1* expression (Supplementary Fig. 7). Treatment with GW4064 normalised the expression of all target genes to levels seen in scrambled controls (Fig. 6c–j, Supplementary Fig. 8). In addition, co-treatment with 22-S HC partially restored the expression levels of *FASN*, *ACC1*, *SREBF1* and *GLUT9*. Additional experiments using the LXRβ antagonist GSK2033 (100 nM, 24 h) resulted in partial rescue of the expression levels of *FASN*, *ACC1* and *SREBF1*, but had no impact on the induction of expression of the other genes caused by *AKR1D1* knockdown (Fig. 6c–j, Supplementary Fig. 8). Taken together, these data provide strong evidence that the observed metabolic effects following genetic manipulation of AKR1D1 are mediated by impaired FXR activation and, to a lesser extent, increased LXR activation.

### 3.7 *AKR1D1* knockdown fuels cellular inflammation

**Fig. 7:**
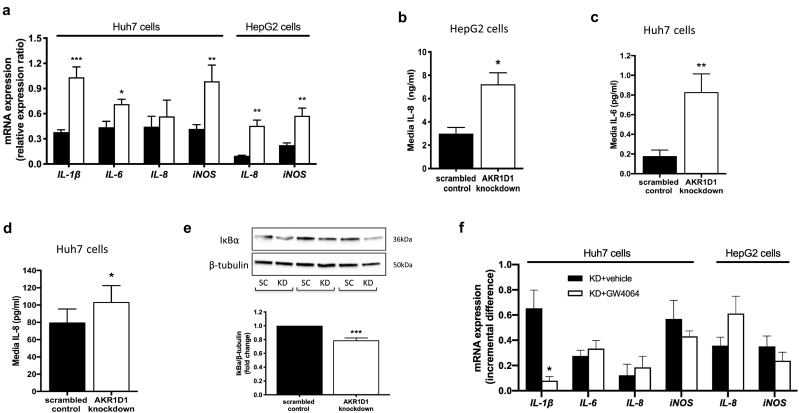
Inflammatory response in human hepatoma cell lines, following AKR1D1 knockdown. *AKR1D1* knockdown (white bars) increased the mRNA expression of pro-inflammatory cytokines IL-1β, IL-6 and IL-8 as did the expression of iNOS in Huh7 and HepG2 cells, as measured by qPCR (a). *AKR1D1* knockdown increased the cell media concentrations of IL-8 in HepG2 cells (b), as well as of IL-6 and IL-8 cell media levels in Huh7 cells (c and d). In addition, *AKR1D1* knockdown decreased the protein expression levels of IκBα, the endogenous inhibitor of NF-κB, which co-ordinates the cellular inflammatory response (e). Pharmacological manipulation of the bile acid receptor FXR, using the FXR agonist GW4064 (5 μM, 24 h), normalised the expression of *IL*-*1β* in Huh7 cells, only, whilst it had no effect on the expression of *IL*-*6*, *IL*-*8* or *iNOS* in either Huh7 or HepG2 cells (f). qPCR data were normalised to 18SrRNA, protein levels were normalised to β-tubulin and cell media levels were corrected to total protein. Representative Western blot images are shown from 3 biological replicates, however formal quantification was performed in n = 7–9 replicates. Data are presented as mean ± se of n = 5–8 experiments, performed in triplicate, *p < 0.05, **p < 0.01, ***p < 0.001, compared to scrambled controls (black bars).

Inflammation is a key pathogenic process in the natural history and progression of NAFLD. In HepG2 cells, *AKR1D1* knockdown increased the mRNA expression of the pro-inflammatory cytokine IL-8, as well as increased IL-8 secretion into the cell culture media. In addition, *AKR1D1* knockdown increased the mRNA expression of inducible nitric oxide synthase (*iNOS*), which is induced in response to an inflammatory insult and has been implicated in the pathogenesis of NAFLD [33] (Fig. 7a and b). Consistent with these findings, *AKR1D1* knockdown increased the mRNA expression of *iNOS* and the expression of the pro-inflammatory cytokines IL-1β and IL-6 in Huh7 cells (Fig. 7a), alongside increases of both IL-6 and IL-8 secretion in the cell culture media (Fig. 7c and d). Furthermore, protein levels of IκBα (nuclear factor of kappa light polypeptide gene enhancer in B-cells inhibitor-α), the endogenous inhibitor of NF-κB that co-ordinates the cellular inflammatory response, were reduced by *AKR1D1* knockdown in Huh7 cells (Fig. 7e).

In order to identify the mechanisms that were responsible for the regulation of the inflammatory response caused by *AKR1D1* knockdown, both HepG2 and Huh7 cells were treated with GW4064 (5μΜ, 24 h) or vehicle, and pro-inflammatory cytokine mRNA expression were explored. In Huh7 cells, GW4064 restored the expression levels of *IL*-*1β* only, but had no impact on the induction of expression of *IL*-*6*, *IL*-*8* or *iNOS* caused by *AKR1D1* knockdown. Similarly, GW4064 failed to restore cytokine mRNA expression in HepG2 cells, suggesting, in total, that the inflammatory response caused by *AKR1D1* knockdown is not entirely FXR mediated (Fig. 7f).

## 4 Discussion

We have provided the first evidence to suggest that AKR1D1 expression is down-regulated across the spectrum of NAFLD and that this may be crucial in the pathogenesis of the condition. Our *in vitro* experiments have shown that limiting *AKR1D1* expression and activity regulates fundamental metabolic processes within human hepatoma cells that govern hepatic insulin sensitivity, lipid accumulation and carbohydrate metabolism. This leads to a net increase in hepatocyte lipid accumulation and an enhanced inflammatory response.

Consistent with our previous study [9], AKR1D1 is able to regulate glucocorticoid availability in human hepatoma cells, revealing the presence of an additional pathway for intracellular steroid hormone metabolism within human liver. In healthy human hepatocytes, glucocorticoids can also be metabolised by a variety of other enzymes, including HSD11B1, SRD5A1 and CBR1. However, considering the lack of expression of HSD11B1 and CBR1 in human hepatoma cells, the potential contribution of these enzymes in cortisone metabolism, resulting in differences in the proportion of the 5β-reduced product, may be small, but cannot be excluded.

Lipid accumulation within hepatocytes is the initial, pre-requisite step in the development of NAFLD. Fatty acid delivery from adipose tissue lipolysis, arising as a consequence of adipose tissue insulin resistance, remains the predominant source of TAG within hepatocytes, however, TAG synthesis *via* DNL increases in importance with NAFLD severity [34,35]. It is plausible that enhanced glucose uptake with *AKR1D1* knockdown may have fuelled the increase in DNL that we observed. Glucose is the most abundant substrate used for DNL. In addition, there is clear evidence that limitation of β-oxidation of fatty acids within hepatocyte mitochondria fuels further TAG accumulation [36]. AKR1D1 knockdown had a dual effect on DNL and β-oxidation. However, as there is an inverse correlation between hepatic DNL and β-oxidation, where increased malonyl-CoA production leads to decreased oxidation *via* CPT1 inhibition [37], it remains unclear whether the observed decrease in β-oxidation is directly attributable to *AKR1D1* knockdown.

In humans, glucose homeostasis is tightly regulated, and abnormalities in hepatic glucose transport have been linked to NAFLD as well as the development of hepatocellular carcinoma [38,39]. Although GLUT2 is the major glucose transporter in primary human hepatocytes [40], GLUT1 and GLUT9 are the major contributors to glucose influx in hepatoma cells [41]. Gene silencing of *AKR1D1* led to increased glucose flux, with changes in GLUT1 and GLUT9 mRNA expression and reduced cell media glucose levels, suggesting increased glucose transport into the cells. Increased glucose transport and changes in glycogen phosphorylase are likely to underpin the increase in glycogen we observed. Matching our mechanistic findings, increased intracellular hepatic glycogen content has been described in obese non-diabetic and T2DM patients [42,43].

The mechanisms underpinning our observations are complex, and do not appear to be related to alterations in glucocorticoid availability; cortisol failed to regulate most of the genes that were regulated as a consequence of *AKR1D1* knockdown. In addition, cell treatments with CA had no effect on gene expression either; however, additional CDCA treatments prevented the induction of genes involved in lipid and carbohydrate metabolism and insulling signalling, following *AKR1D1* knockdown. In this regard, the higher potency of CDCA, compared to CA, to activate FXR is well described [44,45], and our data suggested that the observed changes are the result of alterations in CDCA production and FXR activation. FXR activation represses the activity of genes involved in TAG synthesis, including inhibition of the expression of SREBP-1c and *FASN* [46,47]. Consistent with these findings, *AKR1D1* knockdown increased lipogenic gene expression and these changes was abolished following GW4064 treatment, suggesting that the effects of *AKR1D1* knockdown on lipid metabolism are largely, although not exclusively, driven by FXR activation.

The role of FXR in the pathogenesis of metabolic disease and in particular NAFLD is still not fully understood. Genetic deletion of FXR expression has shown a detrimental impact on lipid profiles, insulin sensitivity and hepatic steatosis [48,49]. However, these models are complex, with compensatory increases in bile acid production rates, which also have the potential to impact the metabolic phenotype through activation of alternative bile acid receptors, including TGR5, in a tissue-specific manner.

At a cellular level, FXR activation supresses SREBP-1c expression in mice [50], an action that is mediated through small heterodimer partner (NR0B2/SHP). In our study, although FXR agonism restored *SREBF1* mRNA expression in the *AKR1D1* knocked down HepG2 cells, no differences were observed in scrambled control-transfected HepG2 cells, following GW4064 treatment (Supplementary Fig. 7f). Therefore, potential differences in the regulation of *SREBF1* by FXR activation between rodents and humans cannot be excluded. In parallel, FXR activation also increases fatty acid oxidation within hepatocytes through induction of PPARα [51]. FXR has an additional role in regulating carbohydrate metabolism although some of the published data have been conflicting; FXR agonists can suppress gluconeogenesis and increase glycogen synthesis [52,53], whilst FXR null mice have fasting hypoglycaemia with decreased hepatic expression of gluconeogenic enzymes as well as decreased hepatic glycogen content [54,55].

If limiting FXR activation by *AKR1D1* knockdown was the only mechanism responsible for our observations, some of our data would conflict with the published literature. Obeticholic acid (semi-synthetic bile acid analogue) treatment of human liver slices increased the expression of both SLC51A and B [56], and this mirrors the observations we made in our *AKR1D1* knockdown experiments. An explanation for these findings may lie in analysis of the composition of the bile acid pool following *AKR1D1* knockdown. The differing affinities of both primary and secondary bile acids to activate (or antagonize) bile acid receptors are well described [57] and, therefore, this may also offer an explanation as to why our observations are not rescued by simple FXR receptor agonism. Importantly, in patients with NAFLD, whilst total faecal primary bile acid levels are increased in comparison with healthy controls, there is a disproportionate increase in CDCA compared to CA [18] although in this study, circulating bile acid levels were not measured.

The role of LXR receptor activation to regulate lipogenic gene expression, including *SREBF1*, *FASN*, *ACC1* and *SCD1*, has been well described [[58], [59], [60]]. In our study, treatment with the LXRα antagonist 22-S hydroxycholesterol reduced the effect of *AKR1D1* knockdown on lipogenic gene expression, although to a lesser extent when compared to FXR agonism. The role of LXRβ in hepatic lipogenesis has been conflicting. A study from Repa et al. [61] revealed reduced lipogenic gene expression in LXRα, but not LXRβ, deficient mice; in contrast, in a more recent study, LXβ deficiency reduced the effects of the synthetic LXR ligand T0901317 on lipogenic gene expression in mice [62]. In our work, LXRβ antagonism partially restored the effects of *AKR1D1* knockdown on lipogenic gene expression, highlighting, in total, the additive role of LXRs in the metabolic phenotype observed following *AKR1D1* knockdown.

Inflammation and liver injury are hallmarks in NAFLD/NASH; lipid accumulation within hepatocytes has also been proposed to trigger the activation of inflammatory pathways [63]. Further insight in NAFLD pathophysiology has implicated bile acids in the prevalence of inflammation with increased generation of toxic hydrophobic bile acids that result in cholestatic, inflamed livers. In this work, genetic manipulation of AKR1D1 expression and activity regulated the inflammatory response in human hepatoma cells by enhancing the expression and secretion of pro-inflammatory cytokines alongside activation of the NF-κB pathway. In line with this, a significant number of studies have linked cholestasis and consequent liver inflammation and dysfunction with mutations in AKR1D1 [[64], [65], [66]]. It is thought that these mutations lead to increased levels of bile acid precursors, allo-bile acids (bile acids produced *via* 5αR activity) and low levels of CDCA (due to 27-hydroxylase activity and induction of the alternative pathway), which can then drive liver injury.

There is increasing evidence on the role of FXR as a regulator of cellular inflammation and immune response; activation of the receptor induces the expression of anti-inflammatory genes [67] as well as suppressing NF-κB activation by negative regulation of inflammatory gene expression in the liver [68]. GW4064 has been reported to decrease hepatic inflammation, both *in vitro* and *in vivo*, by reducing the expression of pro-inflammatory cytokines [69,70]. In our study, GW4064 restored IL-1β mRNA expression in Huh7 cells, only. This finding, in combination with the observed IκBa degradation caused by *AKR1D1* knockdown, suggests that AKR1D1 regulates inflammatory response in a both NF-κB-dependent and NF-κB-independent way of action. (Supplementary Fig. 9).

## 5 Conclusions

In conclusion, we have shown that *AKR1D1* is down-regulated across the spectrum of NALFD, and that this drives a detrimental impact on hepatocyte lipid accumulation and inflammation. Further studies are clearly warranted to explore both the mechanisms by which this occurs as well as the potential to manipulate activity to alter NAFLD progression.

## Author contributions

Study development, N.N., L.L.G. and J.W.T.; Methodology, N.N., C.G., N.J.D., L.H.; Investigation, N.N., L.M., S.A., C.G., M.v.d.B., C.M., B.S., R.S.G., A.A., B.A.H.; Writing - Original draft, N.N., S.A., J.W.T.; Writing - Review & Editing, N.N., W.A., H.U.M., T.P., J.R., L.H., J.W.T.; Supervision, W.A., L.H., J.W.T.; Funding Acquisition, J.W.T.

## Funding and acknowledgements

This work was supported by the Medical Research Council (programme grant to JWT ref. MR/P011462/1, unit support to RDC MC_U142661184); NIHR Oxford Biomedical Research Centre (principal investigator award to JWT); British Heart Foundation (senior fellowship to LH ref. FS/15/56/31645); National Institute of Environmental Health Sciences (P30-ES013508 awarded to TMP); NIHR Birmingham Biomedical Research Centre (Principal investigator award to W.A.). The views expressed are those of the author(s) and not necessarily those of the NHS, the NIHR or the Department of Health or the National Institute of Environmental Health.

## Declaration of Competing Interest

Nothing to declare. TMP is a consultant for Research Institute for Fragrance Materials, is a recipient of a sponsored research agreement from Forendo, and is founding director of Penzymes, LLC.

## Appendix A Supplementary data

Fig. 1 AKR1D1 expression in patients with advanced NAFLD, compared to patients with mild NAFLD, as extracted from publicly available databases (GEO accession GSE49541). Fig. 2 AKR1D1 mRNA expression levels in human hepatoma cell lines and in primary human hepatocytes (a). *AKR1D1* knockdown decreased cortisone clearance (b) and 5β-THE generation (c) after 8h of cortisone treatment, in HepG2 cells. Furthermore, AKR1D1 mRNA and protein levels increased, following *AKR1D1* over-expression in HepG2 cells (d and e). *AKR1D1* over-expression (white bars) increased cortisone clearance (f) and tetrahydrocortisone generation [5β-THE] (g), after 8h of cortisone treatment, in HepG2 cells, compared to no cell controls (grey bars) or vector only transfected cells (black bars). qPCR data were normalised to 18SrRNA. Data are presented as mean±se of n=3 experiments, performed in triplicate, *p<0.05, **p<0.01, ***p<0.001, compared to empty vector transfected controls. Fig. 3 In HEK293 cells, AKR1D1 mRNA and protein expression increased, following *AKR1D1* over-expression (a and b), with a subsequent increase in cortisone clearance (c) as well as 5β-THE generation (white bars) (d), after 8h of cortisone treatment. qPCR data were normalised to 18SrRNA. Data are presented as mean±se of n=5 experiments, performed in triplicate, *p<0.05, **p<0.01, ***p<0.001, compared to empty vector transfected controls. Fig. 4 RNAseq statistics for the various genes looked throughout the manuscript validate the qPCR results, both on a p value level and direction of effect, following *AKR1D1* knockdown in HepG2 cells. Fig. 5 Similar to HepG2 cells, *AKR1D1* knockdown increased the mRNA expression levels of lipogenic synthesis genes in Huh7 cells (a). In addition, *AKR1D1* knockdown increased total ACC protein levels (b) and TAG accumulation (c) in Huh7 cells. Western blotting pictures demonstrate results from 3 representative biological replicates. Protein data were normalised to β-tubulin. Data are presented as mean±se of n=5-6 experiments, performed in triplicate, *p<0.05, **p<0.01, compared to scrambled controls (black bars). Fig. 6 *AKR1D1* over-expression (white bars) had no effect on the mRNA expression of carbohydrate metabolism genes (a), lipogenic genes (b) or TAG accumulation (c), in HepG2 cells. qPCR data were normalised to 18SrRNA. Data are presented as mean±se of n=5 experiments, performed in triplicate, compared to scrambled controls (black bars). Fig. 7 In HepG2 cells, cortisol treatment (500nM, 24h) did not change the expression of *CYP7A1* (a) or the expression of genes *FASN*, *GLUT9*, *ACC1*, *SCD1*, *SREBF1* and *AKT1* (b-h). In contrast, pharmacological manipulation of the bile acid receptor FXR, using the FXR agonist GW4064 (5μM, 24h), reduced the expression of *CYP7A1 and GLUT1* as well as the expression of the lipogenic genes *FASN*, *ACC1* and *SCD1* (a-e). In addition, treatment with the LXRα antagonist 22-S Hydroxycholesterol (22-S HC) (10μM, 24h) increased the expression of *GLUT9* and decreased the expression of *SREBF1* and *SCD1* (c, f, g). qPCR data were normalised to 18SrRNA. Data are presented as mean±se of n=6 experiments, performed in triplicate, compared to scrambled controls (black bars). Fig. 8 Expression of *CYP7A1* (a), *FASN* (b), *ACC1* (c), *SREBF1* (d), *SCD1* (e) and *AKT1* (f), following GW4064 (5μΜ, 24h), 22-S HC (10μΜ, 24h) and GSK2033 (100nM, 24h) treatment in *AKR1D1* knockdown HepG2 cells, as measured by qPCR. Data are expressed as mean±se of of n=6 experiments, *p<0.05, **p<0.01, ***p<0.001, compared to scrambled controls (black bars). Fig. 9 Proposed *in vitro* role of AKR1D1 knockdown in human hepatocytes. (1): Steroid hormones and cholesterol are metabolised by AKR1D1. AKR1D1 silencing resulted in (2): disturbed bile acid synthesis and decreased clearance of steroid hormones. Disturbed bile acid synthesis resulted in (3): Increased TAG accumulation, decreased fatty acid oxidation and (4): unusual/toxic bile acids production. The proposed mechanism suggests that increased TAG accumulation, unusual bile acids production or both resulted in (5): increased expression of pro-inflammatory cytokines, with a subsequent (6): increase in IL-6 and IL-8 secretion in the cell culture media. Image 1
