# Supplementary material for: AKR1D1 is a novel regulator of metabolic phenotype in human hepatocytes and is dysregulated in non-alcoholic fatty liver disease
Source: Metabolism. 2019 Oct;99:67–80. doi: 10.1016/j.metabol.2019.153947 (PMC6744372; doi:10.1016/j.metabol.2019.153947)

Supplementary figure 1

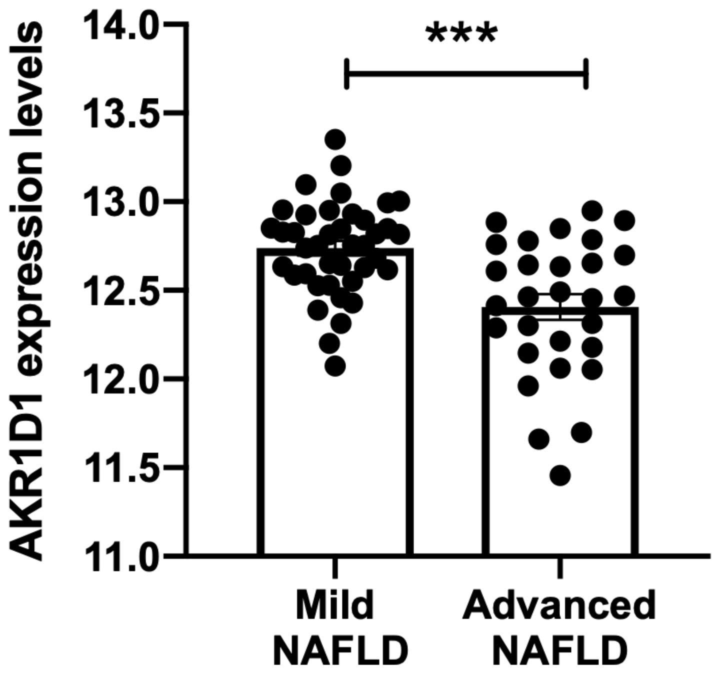

Supplementary figure 2

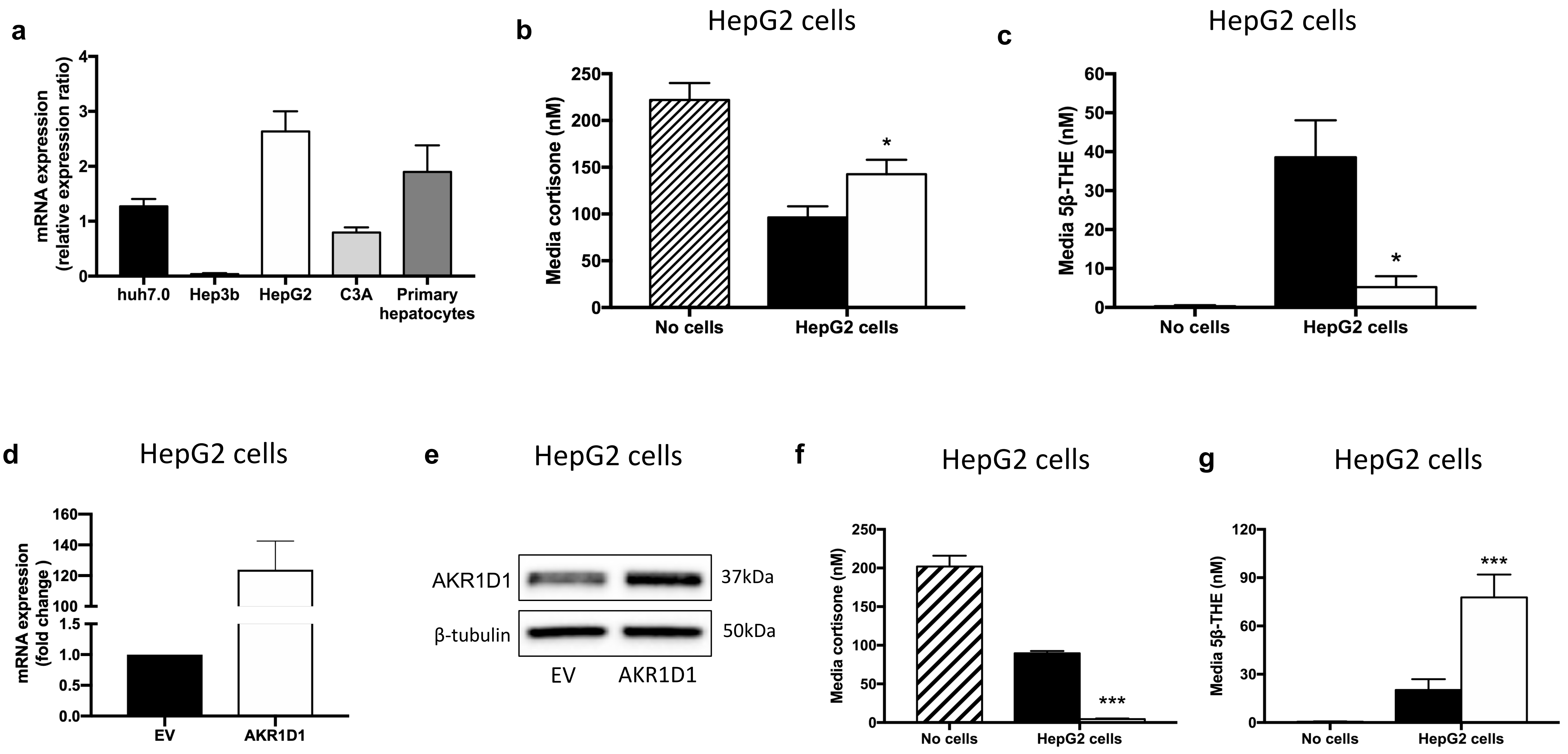

Supplementary figure 3

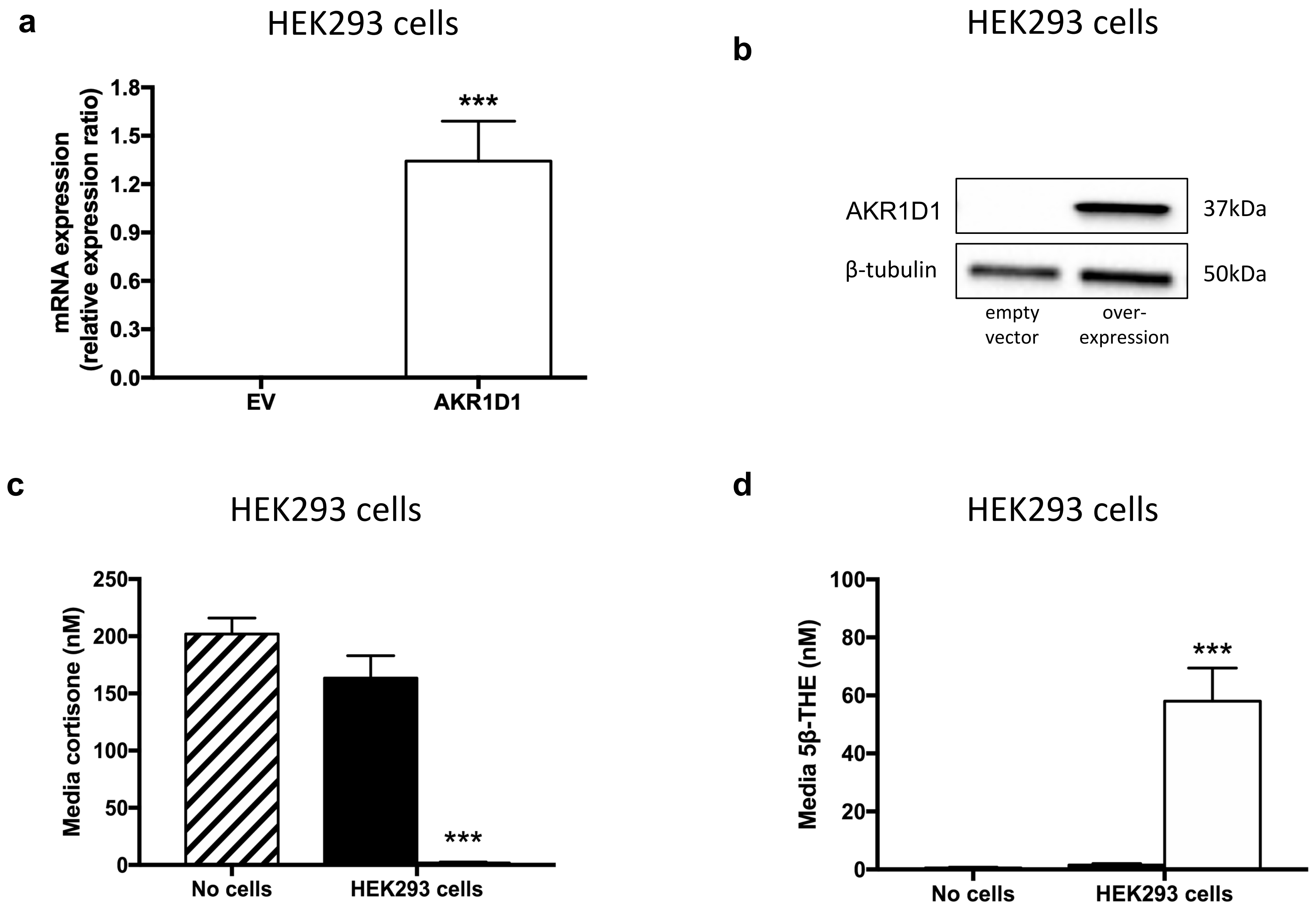

Supplementary figure 4

Gene	LogFC	FDR-adjusted P value
SLC2A1	1.17	1.89×10^{-2}
GAPDH	-0.30	2.93×10^{-1}
CYP7A1	3.49	2.44×10^{-3}
FASN	0.95	1.49×10^{-2}
ACACA (ACC1)	0.74	2.40×10^{-2}
PPARA	-0.52	2.82×10^{-2}
SCD	0.61	3.49×10^{-2}
ABCA1	-0.86	9.11×10^{-3}
PYGL	-0.61	3.22×10^{-2}
SREBF1	0.57	2.23×10^{-2}

Supplementary figure 5

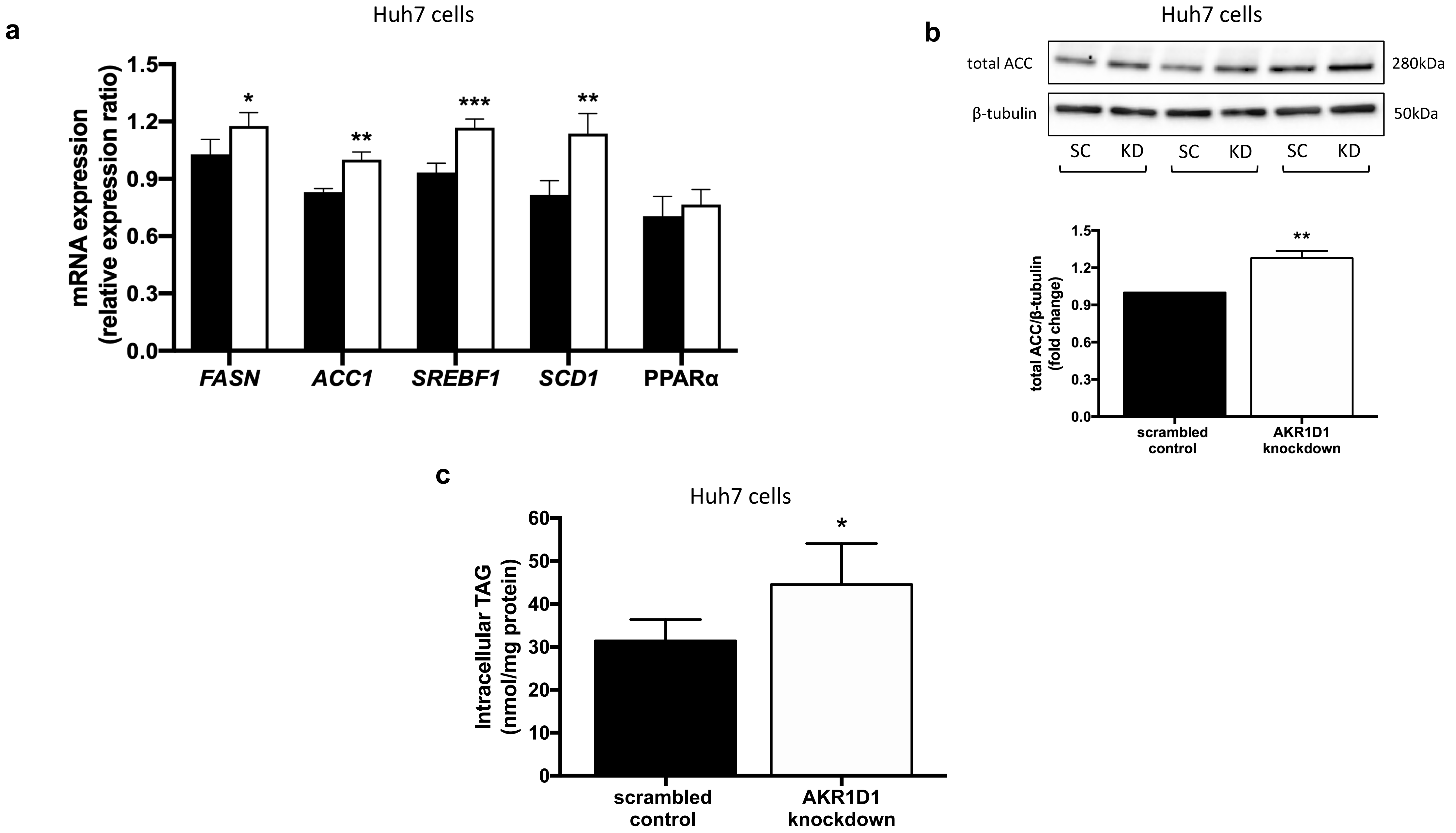

Supplementary figure 6

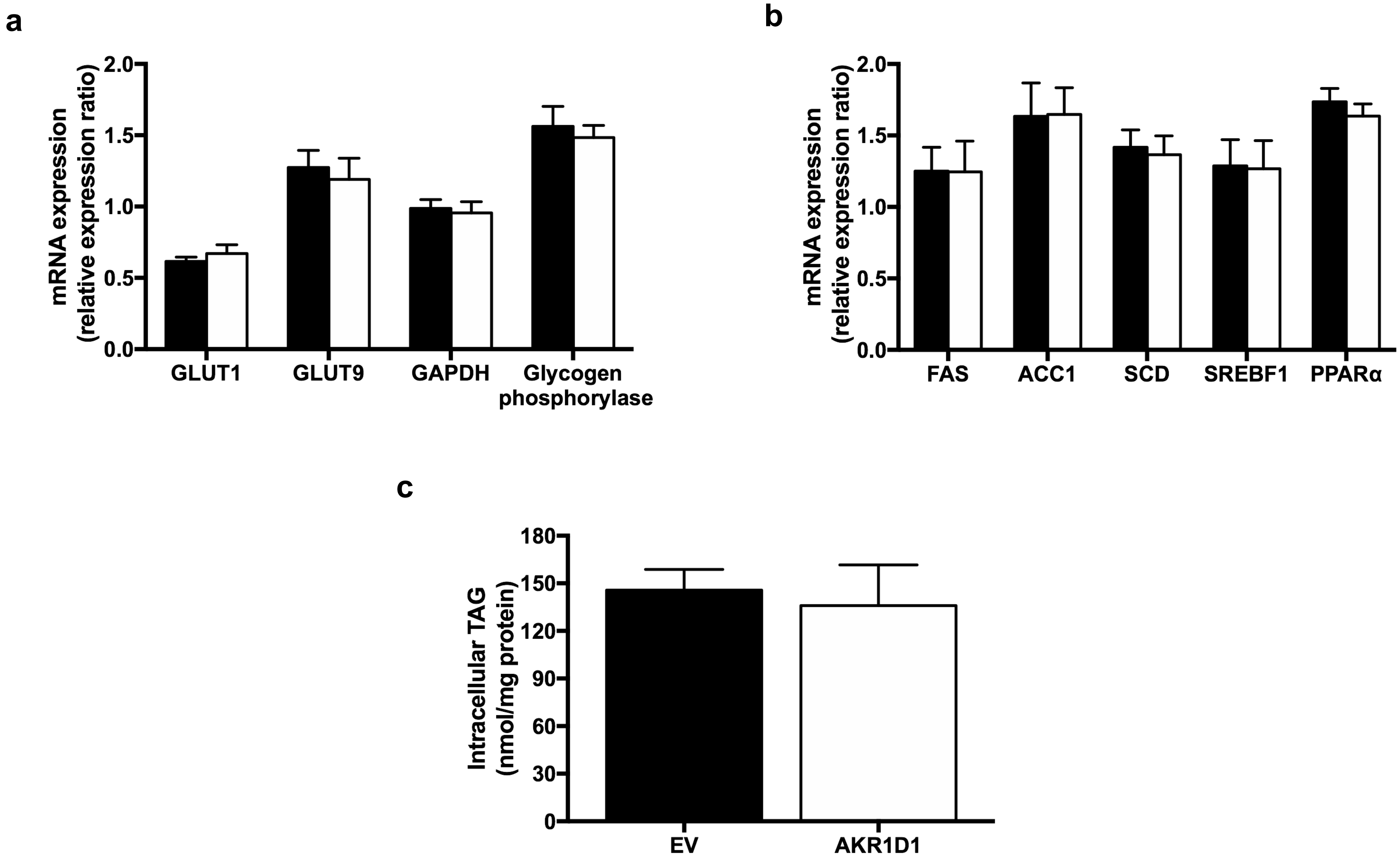

Supplementary figure 7

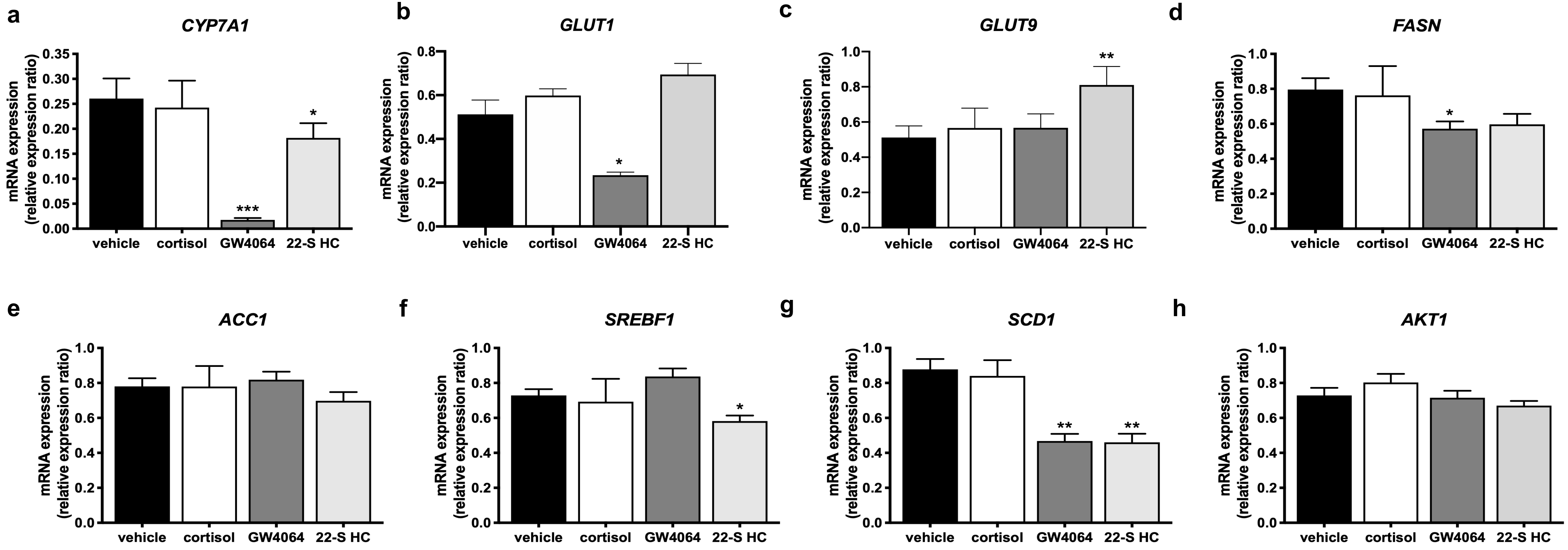

Supplementary figure 8

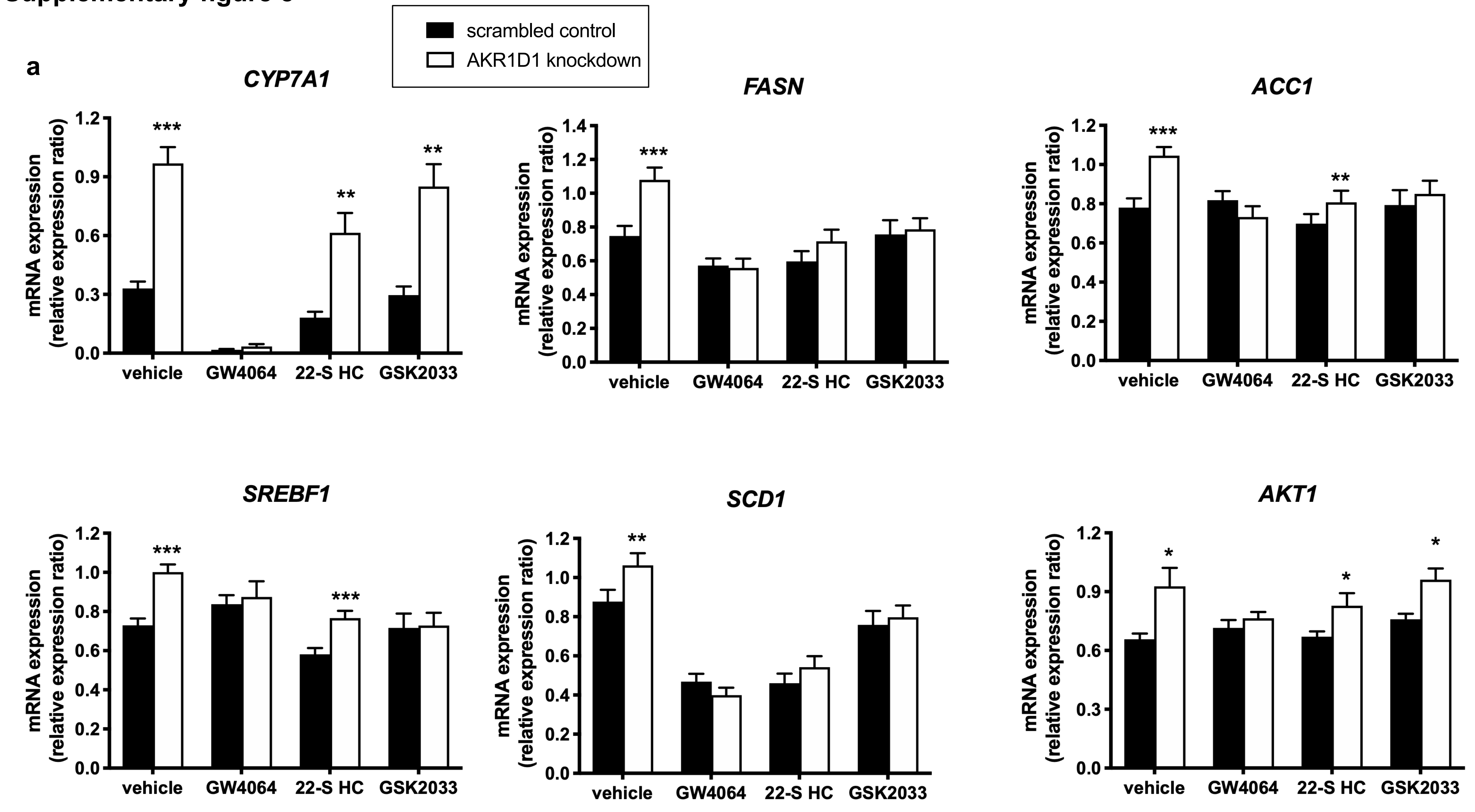

Supplementary figure 9

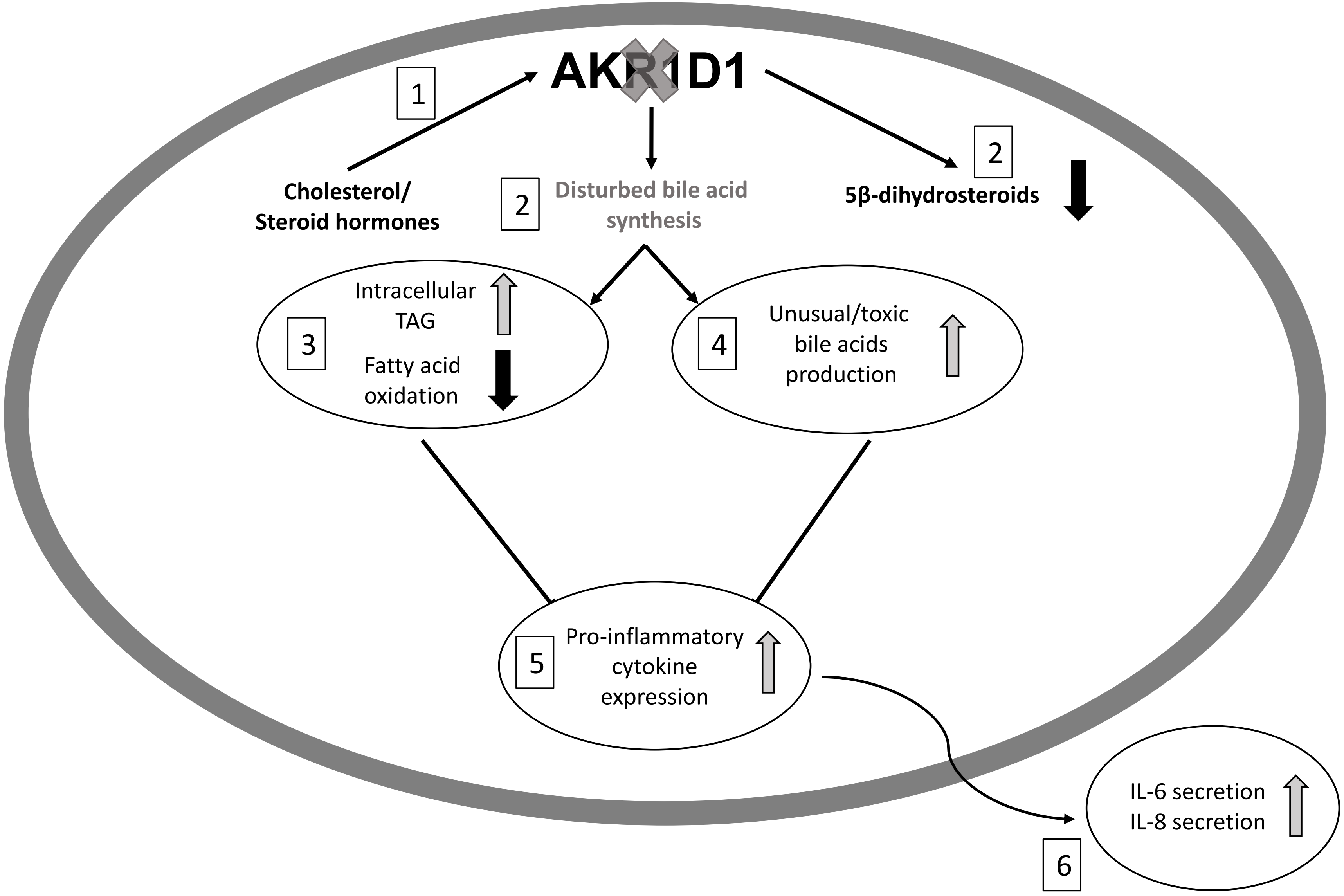

Supplement: Supplementary file 1 — Fig. 1 AKR1D1 expression in patients with advanced NAFLD, compared to patients with mild NAFLD, as extracted from publicly available databases (GEO accession GSE49541). Fig. 2 AKR1D1 mRNA expression levels in human hepatoma cell lines and in primary human hepatocytes (a). AKR1D1 knockdown decreased cortisone clearance (b) and 5β-THE generation (c) after 8h of cortisone treatment, in HepG2 cells. Furthermore, AKR1D1 mRNA and protein levels increased, following AKR1D1 over-expression in HepG2 cells (d and e). AKR1D1 over-expression (white bars) increased cortisone clearance (f) and tetrahydrocortisone generation [5β-THE] (g), after 8h of cortisone treatment, in HepG2 cells, compared to no cell controls (grey bars) or vector only transfected cells (black bars). qPCR data were normalised to 18SrRNA. Data are presented as mean±se of n=3 experiments, performed in triplicate, *p<0.05, **p<0.01, ***p<0.001, compared to empty vector transfected controls. Fig. 3 In HEK293 cells, AKR1D1 mRNA and protein expression increased, following AKR1D1 over-expression (a and b), with a subsequent increase in cortisone clearance (c) as well as 5β-THE generation (white bars) (d), after 8h of cortisone treatment. qPCR data were normalised to 18SrRNA. Data are presented as mean±se of n=5 experiments, performed in triplicate, *p<0.05, **p<0.01, ***p<0.001, compared to empty vector transfected controls. Fig. 4 RNAseq statistics for the various genes looked throughout the manuscript validate the qPCR results, both on a p value level and direction of effect, following AKR1D1 knockdown in HepG2 cells. Fig. 5 Similar to HepG2 cells, AKR1D1 knockdown increased the mRNA expression levels of lipogenic synthesis genes in Huh7 cells (a). In addition, AKR1D1 knockdown increased total ACC protein levels (b) and TAG accumulation (c) in Huh7 cells. Western blotting pictures demonstrate results from 3 representative biological replicates. Protein data were normalised to β-tubulin. Data are presented as mean [file mmc1.pdf]
